# SARS-CoV-2 Infection and Possible Neonatal Neurological Outcomes: A Literature Review

**DOI:** 10.3390/v14051037

**Published:** 2022-05-13

**Authors:** Flávia Maciel de Moraes, Julia Werneck Paulino Soares de Souza, Letícia Pires Alves, Milena Ferreira Ribeiro de Siqueira, Ana Paula Aguiar dos Santos, Mariana Monteiro de Carvalho Berardo, Marcelo Gomes Granja, Hugo Caire de Castro-Faria-Neto

**Affiliations:** 1 Programa de Pós-Graduação em Biologia Molecular e Celular, Universidade Federal do Estado do Rio de Janeiro—UNIRIO, Rua Frei Caneca, 94-Centro, Rio de Janeiro 20211-010, Brazil; flaviamdemoraes.1997@gmail.com (F.M.d.M.); le-alves98@hotmail.com (L.P.A.); 2 Instituto de Educação Médica—IDOMED, Escola de Medicina, Universidade Estácio de Sá, Avenida Presidente Vargas, 1121-Centro, Rio de Janeiro 20071-004, Brazil; juliawerneckp@gmail.com (J.W.P.S.d.S.); marianamcberardo@gmail.com (M.M.d.C.B.); 3 Faculdade de Biomedicina, Instituto Biomédico, Universidade Federal Fluminense, Rua Ernani Melo, 101-São Domingos, Niterói 24210-130, Brazil; milenafrs@id.uff.br; 4 Escola de Medicina, Fundação Técnico-Educacional Souza Marques, Avenida Ernani Cardoso, 335-Cascadura, Rio de Janeiro 20020-080, Brazil; anapagsantos@gmail.com; 5 Laboratório de Imunofarmacologia, Fundação Oswaldo Cruz-Fiocruz, Avenida Brasil, 4365-Manguinhos, Rio de Janeiro 21045-900, Brazil; hugocfneto@gmail.com

**Keywords:** SARS-CoV-2, neurological outcomes, neonatal infections, brain

## Abstract

The virus responsible for COVID-19 is designated “severe acute respiratory syndrome coronavirus 2” (SARS-CoV-2), a highly transmissible and pathogenic coronavirus. Although people of all ages are susceptible to SARS-CoV-2 infection, clinical manifestations may vary with age. The response of neonates to SARS-CoV-2 infection or exposure differs from that of children and adults. Encephalitis due to viral infections in the central nervous system (CNS) and childhood multisystem inflammatory syndrome (MIS-C) are some of the possible neonatal consequences of SARS-CoV-2 infection. This review aims to verify possible neonatal neurological outcomes after SARS-CoV-2 infection. Overall, the cellular and molecular basis of the neurological sequelae of SARS-CoV-2 in neonates remains unclear, and attempts to elucidate the pathophysiology of COVID-19 involve a comparison with the mechanism of other viral diseases. There are a considerable number of case reports in the literature exploring neurological outcomes in the neonatal period. In this review, we present possible effects of SARS-CoV-2 in neonates, emphasizing the importance of monitoring this group. The mechanisms of SARS-CoV-2 entry into the CNS have not yet been fully elucidated, and the potential severity of SARS-CoV-2 infection in neonates, as well as the possible short- and long-term neurological sequelae, remain unclear.

## 1. Introduction

Coronaviruses are a group of viruses that can cause infection in many different animals, leading to mild-to-severe respiratory infections in humans [1]. The virus that causes COVID-19 is severe acute respiratory syndrome coronavirus 2 (SARS-CoV-2) [2]. SARS-CoV-2 is a highly transmissible and pathogenic coronavirus identified in late 2019 that has caused a pandemic of acute respiratory disease, which has impacted human health and public safety [1].

People of all ages are susceptible to SARS-CoV-2 infection, though clinical manifestations may vary with age. Older people (over 60 years old) with comorbidities are more likely to develop severe respiratory disease requiring hospitalization, whereas most young people and children have only mild disease or are asymptomatic [1].

Neonates differ from other at-risk groups due to their possible route of exposure to the virus: although neonates can contract SARS-CoV-2 through close personal contact in much the same way as other groups, some studies have considered the possibility of vertically acquired infection. Moreover, the response of newborns to infection is different from that of older children and adults; consequently, neonates might be more prone to infection and severe disease [3]. The etiology of the reduced infection rate and weakened immune response to infection in newborns and older children has been a matter of debate and remains uncertain. Explanations include changes in ACE2 expression patterns with age and evolution of the immune system and responses to infection that occur with development [4]. In general, the literature concerning the severity of the disease in neonates is controversial. Gale et al. (2020) [3] and Barrero-Castillero et al. (2020) [4] suggested that newborns may be at a greater risk of experiencing severe illness than older children, whereas Stafstrom et al. (2020) [5] indicated that neonates may be less likely to become infected and develop severe symptoms.

Although a quarter of neonates infected with SARS-CoV-2 are asymptomatic, most case reports of COVID-19 in newborns indicate that they are infected postnatally and may present gastrointestinal and respiratory symptoms. Among those who are symptomatic, the most common clinical presentations are respiratory distress, fever, and food intolerance [4]. They may also manifest atypical signs and symptoms, such as shock, seizures, and electrolyte abnormalities, as opposed to the more common respiratory and gastrointestinal symptoms [6]. Abdominal pain, shallow breathing, vomiting milk, diarrhea, and dry cough are the most common gastrointestinal manifestations in neonatal COVID-19 infection [7].

Furthermore, coronaviruses have been detected in the cerebrospinal fluid (CSF), suggesting that once the pathogen reaches the lungs, it spreads throughout the body, eventually to the central nervous system (CNS) [8]. Coronaviruses have the potential to cause nerve injury via various pathways. The tropism of the virus for the CNS can lead to serious outcomes, including encephalitis, toxic encephalopathy, and severe acute demyelinating disorders [8].

The mechanisms of COVID-19-induced nervous system damage may involve direct infection, blood circulation pathways, neuronal pathways, hypoxia injury, immune injury, and angiotensin-converting enzyme-2 (ACE2)-associated effects, among others. It is currently believed that COVID-19, in association with host immune mechanisms, may cause acute infection to become a sustained process that might lead to neurological damage [8].

In this review, we provide information regarding possible neurological outcomes due to SARS-CoV-2 infection during the neonatal period in addition to the most common symptoms and clinical presentations.

## 2. Methods

We searched PubMed for studies published from 2006 to 2022 using the following medical keywords and terms: “neonatal”, “neonate”, “newborn”, “neonatal infections”, “coronavirus”, “coronavirus disease”, “SARS-CoV-2 infection”, “COVID-19 infection”, “neurological outcomes”, “outcomes”, and “brain”. The search strategy used a combination of standardized terms and keywords as well as manual review of select articles for additional related articles. We selected articles applicable to a general-medicine readership, prioritizing case reports, randomized clinical trials, systematic reviews, and clinical practice guidelines. Exclusion criteria included non-English articles, studies involving non-neurological sequelae, and studies performed on animals.

## 3. Perinatal SARS-CoV-2 Infection and Immune Response in Neonates

Pregnant women are considered a high-risk group and are more likely to need intensive care for COVID-19 than nonpregnant women [9]. Mullins et al. reported 4005 pregnant women with suspected or confirmed SARS-CoV-2 infection in the PAN-COVID registry, which includes pregnancies at any stage with suspected or confirmed maternal SARS-CoV-2 infection, and the AAP-SONPM National Perinatal COVID-19 registry, which includes pregnancies with positive maternal testing for SARS-CoV-2 from 14 days prior to delivery to 3 days after delivery. Neonatal SARS-CoV-2 infection was reported in 2.0% of those with confirmed infection in the PAN-COVID database and in 1.8% of those in the AAP-SONPM database [10]. Khoury et al. (2020) [11] assessed 241 births in women with SARS-CoV-2 infection at five different New York medical centers. Of the 236 live births with documented SARS-CoV-2 test results, 230 (97.5%) tested negative within 24–96 h of life [11]. Kayem et al. (2020) [12] also assessed 617 pregnant women in 33 participating centers who had been diagnosed with COVID-19. Among the 486 (78.8%) women who had recovered from COVID-19, 181 had given birth, with only 2 (1.1%) newborns having a positive SARS-CoV-2 RT–PCR result [12].

The National Registry for Surveillance and Epidemiology of Perinatal COVID-19 Infection (NPC-19) is a collaborative effort of the Section on Neonatal–Perinatal Medicine (SONPM) of the AAP, the Vermont Oxford Network (VON) and MedNAX. The NPC-19 is collecting data on mother–infant dyads with the goal of identifying risk factors for neonatal transmission and outcomes. Data from the National Registry for Surveillance and Epidemiology of Perinatal COVID-19 Infection (NPC-19) showed 91/2498 (3.6%) viral tests to be positive for neonates born to mothers with confirmed SARS-CoV-2 infection [13].

The etiology of the reduced infection rates and the dampened immune response to infection found in newborns remain unclear [14]. ACE2 gene expression is lowest in younger children and increases with age. As ACE2 is the receptor that SARS-CoV-2 uses for host entry, it is possible that the decreased expression of this receptor is, at least partially, responsible for the reduction in the observed infection rates [15].

Most neonatal SARS-CoV-2 infections are acquired after birth via horizontal virus transmission from family members and healthcare workers [15]. Nevertheless, it can be difficult to differentiate vertical from horizontal transmission. To date, the presence of a positive SARS-CoV-2 IgM in a newborn between birth and 7 days of life suggests a fetal response to intrauterine infection, whereas IgM positivity after 7 days suggests early intrapartum or postnatal infection [16,17,18].

### 3.1. Clinical Presentations in Newborns

Documentation of the clinical signs, manifestations, and disease course in the medical literature continues to grow. To evaluate the clinical presentations of SARS-CoV-2 infection in newborns, we selected a group of case reports, case series, and other studies. We analyzed published data for approximately 87 newborns with a positive RT-PCR test for SARS-CoV-2 as well as clinical features; Supplementary Table S1 shows the results (Clinical manifestations in newborns during SARS-CoV-2 infection) [19,20,21,22,23,24,25,26,27,28,29,30,31,32,33,34,35,36,37,38,39,40,41,42,43,44,45,46,47,48,49,50,51,52,53,54,55,56,57,58,59,60,61,62]. We evaluated 45 different articles and case reports for the different clinical manifestations in neonates during SARS-CoV-2 infection. Other variables important for evaluating the severity of the clinical manifestations include neonatal maturity, Apgar score, and outcome (i.e., discharge after recovery, not reported, or death).

Of a total of 87 neonates, 83 were discharged after recovery, 1 neonate outcome was not reported, and 3 neonates died. In relation to the three deaths, all newborns presented respiratory distress and were premature.

The percentage of each clinical manifestation found in those articles is reported in Table 1 (percentage of each clinical presentation during neonatal SARS-CoV-2 infection). In total, 22.99% (*n* = 2) of neonates were asymptomatic. In those with symptoms, respiratory features were primarily cough, tachypnea, coryza, and respiratory distress. Respiratory presentations were most frequent (57.47%), with respiratory distress being most common (45.98%), followed by cough (8.05%). A total of 26.44% of newborns were febrile. Vomiting, intolerance to feeding, and abdominal distension were among the gastrointestinal presentations, present in 21.84% of newborns. The most common gastrointestinal symptom was intolerance to feeding (18.39%). Cardiovascular features were tachycardia and hypotension, which were present in 4.60% of the infants. Neurological features were noted in 26.44% of neonates, with lethargy being the most common symptom (9.20%).

### 3.2. Diagnosis of SARS-CoV-2 in Neonates

Nucleic acid amplification is the most reliable test for diagnosing COVID-19, and the most sensitive test is reverse transcriptase real-time polymerase chain reaction (RT-qPCR) [63]. The technique consists of two PCRs in sequence: RT-PCR followed by real-time PCR for detection of viral RNA [64]. This exam targets different genes of SARS-CoV-2, such as RNA-dependent RNA polymerase (RdRp)/helicase (Hel), spike (S), and nucleocapsid (N) [65]. Another nucleic acid amplification technique is loop-mediated isothermal amplification (LAMP), which amplifies the target sequence efficiently and rapidly under isothermal conditions [63].

The systematic review conducted by Pu et al. (2022) [66] assessed 33 studies involving 9360 suspected cases of SARS-CoV-2 infection. The results showed that the overall pooled sensitivity of RT-PCR was 0.96 (95% CI, 0.93−0.98); that of RT-LAMP was 0.92 (95% CI, 0.85−0.96) [66]. The most reliable test for diagnosing SARS-CoV-2 infection in neonates was also RT-PCR. However, as newborns and infants infected with the virus are usually mildly symptomatic, the sensitivity of the test may be reduced by potential false negatives [64].

## 4. Viral Infections and Long-Term Neonatal Outcomes

Viral infections in the fetus or newborn can lead to significant morbidity and mortality [67]. Accordingly, infectious diseases pose a specific challenge to neonatologists. Although the full spectrum of SARS-CoV-2 infection in newborns has yet to be determined, infection with other viruses has provided indicators that can be applied for the prospective evaluation of individuals with SARS-CoV-2 infection.

As mentioned, the cellular and molecular basis of SARS-CoV-2 neurological outcomes in neonates is not yet fully understood. Supplementary Table S1 lists cases of newborns with COVID-19-associated neurological manifestations, such as hypotonia, hypertonia, lethargy, irritability, and apnea. A total of 26.44% of neonates presented these neurological symptoms, corroborating the hypothesis that COVID-19 affects the brain. Stafstrom et al. (2020) [5] discussed potential mechanisms that might explain COVID-19 neurological outcomes. A potential point of entry for SARS-CoV-2 is the ACE2 receptor in the olfactory epithelium. After entering the cell, the virus induces a massive immune response that might lead to excessive cytokine release. Theoretically, virus particles might enter the CNS through cranial nerve pathways. Upon breaching the CNS, neurological signs and symptoms would likely ensue [5]. Furthermore, the CNS itself expresses ACE2, which has been detected on glial cells and neurons and may offer an explanation for the reported neurological involvement [8].

**Figure 1:**
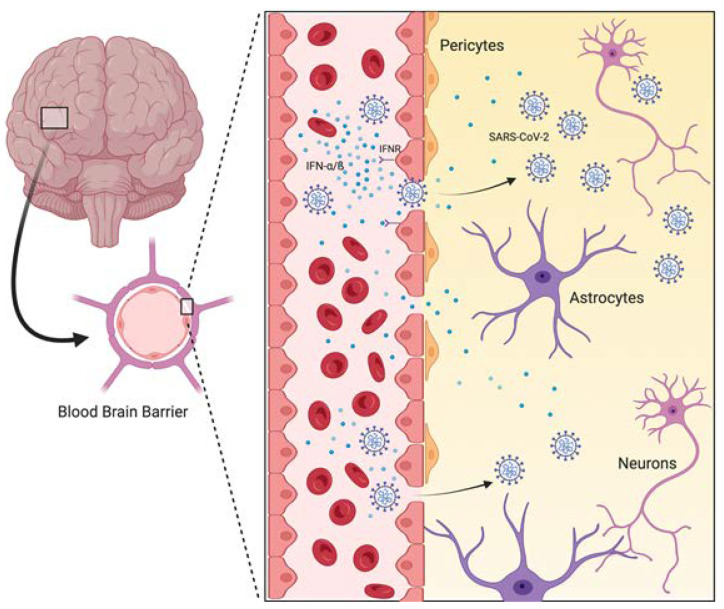
A SARS-CoV-2-infected brain with vessel amplification is depicted. The production of cytokines, such as IFN-α/β and IFNR, initiates the disruption of the blood–brain barrier, after which the virus reaches neurons and astrocytes, initiating an inflammatory process.

Many neurotropic viruses can cross the blood–brain barrier (BBB) and ultimately invade the CNS. For example, herpes simplex virus 1 (HSV-1) can cause encephalitis, leading to cerebral edema and hemorrhage [68]. The brain impairment in this case has been attributed to changes in BBB function, leading to neuroinvasion [69]. In relation to COVID-19, Buzhgygan et al. (2020) [70] suggested that the SARS-CoV-2 spike protein triggers a proinflammatory response in brain endothelial cells. This mechanism may contribute to altered BBB permeability [70]. Furthermore, SARS-CoV-2 infection can invoke a cytokine response and a clear dysregulation of the type-I interferon response, consequently enhancing BBB permeability and tight-junction dysregulation (Figure 1).

Cytomegalovirus (CMV), a DNA virus and a member of the herpes virus family [71], can cause serious neurodevelopmental impairment, including cerebral palsy and sensorineural hearing loss. Although few studies have been conducted to investigate brain injury in congenital CMV infection, Bentz et al. (2006) [72] and Chan et al. (2012) [73] demonstrated that CMV is able to infect different types of leukocytes, enhancing hematogenous dissemination toward the CNS. Additionally, Desforges et al. (2014) [74] showed that other coronaviruses have neuroinvasive capacities because they can enter through the epithelium of the nasopharynx and travel to the CNS [74]. Therefore, SARS-CoV-2 may have a direct neuroinvasive effect that might explain its neurological involvement.

Another mechanism that may explain the neurological outcomes of COVID-19 is infection of human neural progenitor cells by SARS-CoV-2, as demonstrated in brain organoids. Bullen et al. (2020) [75] reported the detection of extensive viral protein expression and viral particles in neuronal progenitor cell populations. The authors employed a human-induced pluripotent stem cell that expresses ACE2 receptors in which virus particles were found in the neuronal cell body after infection.

The literature also reports similar mechanisms for other viral diseases, such as CMV and Zika virus. Mutnal et al. (2011) [76] suggested mechanisms for the pathogenesis of CMV, showing that the main targets of CMV are neural stem cells and neuronal precursor cells within the developing brain. Moreover, neuronal loss was associated with the downregulation of multipotency markers, OCT4 (involved in the self-renewal of embryonic stem cells), and neurotrophins, indicating abnormal brain development.

In September 2015, researchers reported a significant increase in the number of cases of neonatal microcephaly in southeast Brazil concomitant with the Zika virus outbreak (ZIKV) [77]. Brasil et al. (2016) [78] assessed the clinical and imaging findings of 117 neonates born to ZIKV-positive mothers: 42% were found to have significantly abnormal clinical or brain imaging findings, or both, including four infants with microcephaly [78]. Although the pathogenesis remains unclear, one potential mechanism for such microcephaly is that ZIKV triggers apoptosis in neural progenitor cells and attenuates their growth [79].

## 5. Severe Encephalitis with Cytotoxic Brain Edema in a Newborn with COVID-19

Although COVID-19 predominantly affects the pulmonary system, it is a multisystem disease (e.g., gastrointestinal tract, kidneys, liver, and heart). In fact, the involvement of the PNS (peripheral nervous system) and CNS (central nervous system) is being increasingly identified in adults [5]. In contrast, little is known concerning neurological complications related to COVID-19 in newborns. Fragoso et al. (2022) [80] reported a case of a male newborn delivered at 38 weeks of gestation who developed severe encephalitis with cytotoxic brain edema. He tested positive for SARS-CoV-2 on his third day of life and was discharged home due to a lack of symptoms. On his fifth day of life, he was readmitted to the hospital with focal-to-bilateral clonic seizures, predominantly involving his left arm. It was concluded that neonates are at a higher risk of developing seizures during the first week of life due to physiologic aspects such as increased neuronal excitation and decreased inhibition that can lead to long-term neurological sequelae [80]. In addition, the newborn presented lethargy, hypotonia, and brisk tendon reflexes but no primitive reflexes. Owing to these clinical features, the neonate needed to be intubated. Blood tests demonstrated lymphopenia and thrombocytopenia associated with increased D-dimer levels. The authors suggested that his neurological manifestations were caused by a combination of the infection and the immune response, which were responsible for the refractory seizures and cytotoxic brain edema observed.

Overall, neurological implications in neonates with COVID-19 are limited, which may be due to underreporting. Available evidence does not allow for the differentiation between a direct effect of SARS-CoV-2 as a cause of neurological dysfunction and whether symptoms are secondary to an overactivated immune response [5].

### Ischemic Lesions in the Brain

Different clinical findings in a 17-day-old newborn were reported by Brum et al. (2020) [81]. The child was admitted to the hospital emergency room with symptoms of fever for 12 hours, convulsions, and lethargy and presented with consumption coagulopathy, ischemic lesions in the brain, and cardiac involvement. The newborn tested positive for SARS-CoV-2 by RT-PCR, and his parents with no symptoms tested negative [81,82]. A magnetic resonance imaging study was conducted and revealed two small foci of restriction in the left frontal subcortical white matter compatible with acute ischemic lesions. Therefore, central nervous system impairment, ischemic lesions, and coagulopathy may be related to COVID-19 in newborns, with neurological sequelae [81].

## 6. Childhood Multisystem Inflammatory Syndrome (MIS-C)

Childhood multisystem inflammatory syndrome (MIS-C) is a generalized inflammation state accompanied by immune system hyperactivation and probable organ damage. This condition has been linked to SARS-CoV-2 infection in several case reports, mainly in children. The hyperactivation of the immunological system caused by MIS-C can also lead to dangerous consequences [83,84,85]. Fever lasting more than three days, coagulopathy, and hemodynamic instability, as well as gastrointestinal, cardiac, dermatological, renal, respiratory, and neurological symptoms have been reported in individuals with MIS-C. In addition, troponin/elevated NT-proBNP and elevated inflammation markers have been observed in these patients [67,83,84].

One study by Penner et al. (2021) [83] followed patients during infection and for six months post-infection and showed that the most frequent sequelae that persisted after six months were neurological, including proximal myopathy, dysmetria, abnormal saccades, anxiety, and emotional lability [83]. The literature describes headache as the most common neurological feature in MIS-C patients, but other symptoms have also been reported, such as altered mental status, aseptic meningitis encephalitis, seizures, encephalopathy, weakness, ataxia, and dysarthria. Based on current research, systemic inflammation and an exacerbated immune response impact children’s neurodevelopment. Despite the lack of data for neonatal patients with MIS-C, neurological consequences may have an important impact on a neonate’s brain development. During this process, cytokine imbalances at early neurodevelopmental stages can have profound long-term impacts on a variety of disorders, including ASD, schizophrenia, cerebral palsy, depression, and cognitive impairment [86].

### 6.1. Shock and Electrolyte Abnormalities

Electrolyte abnormalities are complications of dialysis that can have both immediate and long-term consequences and increase mortality rates. Since the pandemic began, only a few cases of electrolyte abnormalities and shock have been documented as late-onset COVID-19 outcomes in the neonatal population. Kallimath et al. (2021) [6] described a male newborn who acquired COVID-19 after birth. At only 40 hours of life, the newborn needed to undergo ileal resection and antibiotic therapy due to *Enterococcus faecium* sepsis and intestinal perforation but was discharged on day 16. The subject was admitted again at 26 days old, showing signs of shock such as tachycardia, hypotension, tachypnea, and feeble pulse. Bloodwork revealed electrolyte imbalance with hyperkalemia, hyponatremia, hypocalcemia, hypomagnesemia, and elevated CRP, along with SARS-CoV-2 positivity. He was immediately managed with two crystalloid fluid boules, a dobutamine infusion, and intravenous fluids, in addition to epinephrine for hypotension. On day 2 postadmission to the hospital, the newborn received an infusion of 3% sodium chloride for hyponatremia, sodium bicarbonate, calcium gluconate, and insulin dextrose for hyperkalemia, and potassium binders. After five days, his levels began to normalize, and he was discharged eight days post admission. Daily follow-up was performed for one week via phone calls and in person at 6 and 10 weeks of age, which showed that he had a full clinical recovery and no signs of delayed development [6].

### 6.2. Ophthalmic Manifestations

Ocular manifestations have also been described in newborns. Pérez-Chilman et al. (2021) [87] showed that the virus causes ophthalmologic alterations such as conjunctivitis and intraocular chances. The study was conducted in the Hospital Materno Perinatal Monica Pretelini in Mexico with 15 newborns, 8 females and 7 males. Among the mothers of these newborns, 10 tested positive for SARS-CoV-2. All 15 babies presented with periorbital edema as the most common ophthalmic finding. In addition, chemises and hemorrhagic conjunctivitis were found in 11 newborns. Fundus examination was normal in 7 of the 15 babies; in the other 8, oxygen-induced retinopathy, retinopathy of prematurity, subtle cotton wool spots, and vitreous hemorrhage were found [87].

## 7. SARS-CoV-2 Variants and Potential Differences in Neonates’ Immunological Systems

More than 4000 variants of SARS-CoV-2 have been reported since the start of the pandemic [88]. These variants have been classified by the World Health Organization (WHO) as variants of concern (VOCs) and variants of interest (VOIs). VOCs have a higher transmission rate and are more prone to cause severe disease and/or reduce neutralization by antibodies generated by previous infection or vaccination. Regarding VOIs, they spread less widely but contain mutations similar to those present in VOCs. On 25 January 2022, the WHO updated the list of VOCs, which included five variants, i.e., alpha, beta, gamma, delta, and omicron, and VOIs, comprising lambda and mu [89].

The main target of neutralizing antibodies during SARS-CoV-2 infection is the receptor-binding domain (RBD) region, which belongs to the S1 subunit of the spike protein (glycoprotein responsible for virus entry into host cells). Production of neutralizing antibodies early in the infection is associated with lower levels of virus and better protection against severe disease. These mutations might directly interrelate with the human ACE2 receptor and form part of the epitopes for ACE2-blocking neutralizing antibodies, reducing the effectiveness of the immune protection provided by previous infection and vaccination [88].

The SARS-CoV-2 VOCs and VOIs circulating at present carry various mutations, most of which are in the spike gene [90]. LOTEMPIO et al. (2021) reported a case of a neonate with COVID-19 symptoms who was admitted to the Children’s National Hospital. SARS-CoV-2 RT-PCR detected an extremely high viral rate, and the nonsynonymous amino acid substitution N679S was identified [91]. Data analysis confirmed the presence of the D614G mutation in the viral genome, which is present in the majority of global samples. D614G is an amino acid substitution in the receptor-binding motif (RBM); this mutation modifies the spike protein [92], facilitating the entry of the virus into the host cell. In addition, it is associated with an increase in transmission and infectivity and can be four to nine times more contagious [93,94]. Along with conferring greater resistance to proteolytic cleavage, this mutation causes increased transduction in many cell types, including lung, liver, and colon cells. The combination of S:N679S with S:D614G may contribute to the persistence of the S:N679S variant and neonatal SARS-CoV-2 infection [94].

The main variants, such as alpha, beta, gamma, delta, and kappa, constitute a combination of specific gene mutations that improve transmissibility, virulence, and host immune evasion. Mutations in the protein spike can be classified as RBD, non-RBD, and S1/S2 furin cleavage site mutations; there is also an NSP mutation. Most of the common variants harbor an alteration in the spike protein, changing viral antigenicity and affinity toward ACE2. The alpha variant, for example, bears a combination of mutations that increases the spike protein density of the virion and the binding affinity with ACE2; it can also present lower neutralizing antibody affinity [95].

PHAM et al. (2021) reported a case of a 21-day-old female newborn found to be infected with SARS-CoV-2 variant B.1.1.7 by genomic sequencing. The patient displayed several symptoms of COVID-19, including diarrhea, vomiting, runny nose, and productive cough, which lasted for 3 days. During hospitalization, the patient’s blood tests and vital signs were normal, and she was discharged home after 16 days of care [96].

BOLY et al. (2022) described three premature newborns with very low birth weight (<1500 g); genotype testing was performed for all, confirming SARS-CoV-2 variant B.1.617.2 infection (delta variant). All infants became hyperglycemic and developed transient bone marrow dysfunction following delta variant exposure [97], a variant that appears to affect children to a greater extent than previous variants [98].

## 8. Conclusions

The severity of COVID-19 in neonates and possible neurological outcomes are not yet fully understood. Some researchers suspect that this age group may present with more severe symptoms of the disease, whereas others have suggested that most cases are mild or asymptomatic. Neurological involvement in COVID-19 may be due to access of the virus to the CNS via several mechanisms, subsequently leading to acute neurological symptoms, either directly or through immune dysfunction. The occurrence of long-term medical and neuropsychiatric sequelae is unknown. Therefore, neuropsychological monitoring and research on the long-term outcomes of SARS-CoV-2 infection on neurodevelopment are necessary.

## Figures and Tables

**Table 1:** Clinical features reported in premature and term infants.

Clinical Features	Premature	Term	Total
Asymptomatic	8	12	20
Symptomatic	27	40	67
Fever	3	20	23
Respiratory			
Cough	2	5	7
Respiratory distress	19	21	40
Tachypnea	9	10	19
Coryza	0	4	4
Gastrointestinal			
Vomiting	1	4	5
Intolerance to feeding	5	11	16
Abdominal distension	2	2	4
Neurological			
Lethargy	3	5	7
Irritability	0	3	3
Hypotonia	0	4	4
Apnea	5	1	6
Seizure	0	5	5
Cardiovascular			
Tachycardia	0	4	4

## Data Availability

The data sets used and/or analyzed during the current study are available in the relevant references.

## Supplementary Materials

The following supporting information can be downloaded at: https://www.mdpi.com/article/10.3390/v14051037/s1, Table S1: Clinical manifestations in newborns during SARS-CoV-2 infection.
