# Supplementary material for: SARS-CoV-2 Infection and Possible Neonatal Neurological Outcomes: A Literature Review
Source: Viruses. 2022 May 13;14(5):1037. doi: 10.3390/v14051037 (PMC9143946; doi:10.3390/v14051037)
Supplement: Supplementary file 1 [file viruses-14-01037-s001.zip › viruses-1641572-supplementary.pdf]

Supplementary Table S1. Clinical manifestations in newborns during SARS-CoV-2 infection.

45 different articles and case reports were evaluated, and the data analysis showed the different clinical manifestations in neonates during SARS-CoV-2 infection. Other important variables to evaluate the severity of these clinical manifestations are: Neonatal maturity, Apgar's score and the outcome (Discharge after recovery, not reported or death).

Article Title	Author	Number of neonates	Neonatal maturity	Apgar's score	Neonatal clinical manifestations	Neonatal outcomes
Neonatal COVID-19 Pneumonia: Report of the First Case in a Preterm Neonate in Mayotte, an Overseas Department of France	Abasse et al (2021) (19)	1	Premature	5 at 1 minute<br>7 at 5 minutes	Fever<br>Respiratory distress<br>Tachypnea	Discharge after recovery
Novel coronavirus in a 15-day-old neonate with clinical signs of sepsis, a case report	Aghdam et al (2020) (20)	1	Term infant	Not reported	Fever<br>Lethargy<br>Mottling<br>Tachycardia<br>Tachypnea<br>Respiratory distress<br>Sepsis	Discharge after recovery
Severe COVID-19 during Pregnancy and Possible Vertical Transmission	Alzamora et al (2020) (21)	1	Premature	6 at 1 minute<br>8 at 5 minutes	Cough<br>Respiratory distress	Discharge after recovery

Maternal and perinatal characteristics and outcomes of pregnancies complicated with COVID-19 in Kuwait	Ayed et al (2020) (22)	2	**Neonate 1:**<br>Premature<br>**Neonate 2:**<br>Premature	**Neonate 1**<br>8 at 1 minute<br>9 at 5 minutes<br>**Neonate 2**<br>8 at 1 minute<br>9 at 5 minutes	**Neonate 1**<br>Asymptomatic<br>**Neonate 2**<br>Tachypnea<br>Respiratory distress	Discharge after recovery
Possible Early Vertical Transmission of COVID-19 from an Infected Pregnant Female to Her Neonate: A Case Report	Bandyopadhyay *et al* (2021) (23)	1	Term infant	8 at 1 minute<br>9 at 5 minutes	Asymptomatic	Discharge after recovery
Vertical transmission of COVID-19 in a 1-day-old neonate	Bordbar *et al* (2020) (24)	1	Premature	8 at 1 minute<br>9 at 5 minutes	Respiratory distress<br>Tachypnea<br>Cyanosis<br>Grunting	Discharge after recovery
Neonatal Late Onset Infection with Severe Acute Respiratory Syndrome Coronavirus 2	Buonsenso et al (2020) (25)	1	Term infant	9 at 1 minute<br>10 at 5 minutes	Asymptomatic	Discharge after recovery
Case Report of Severe COVID-19 Pneumonia in a Term Newborn	Cakir *et al* (2021) (26)	1	Term infant	Not reported	Fever<br>Intolerance to feeding<br>Respiratory distress<br>Tachypnea<br>Tachycardia	Discharge after recovery

Pre-labor anorectal swab for SARS-CoV-2 in COVID-19 pregnant patients: is it time to think about it?	Carrosso et al (2020) (27)	1	Term infant	9 at 1 minute<br>10 at 5 minutes	Asymptomatic	Discharge after recovery
Late-Onset Neonatal Sepsis in a Patient with Covid-19	Coronado Munoz et al (2020) (28)	1	Term infant	Not reported	Fever<br>Intolerance to feeding<br>Respiratory distress<br>Pneumothorax<br>Tachypnea, tachycardia, hypothermia and hypotension<br>Coryza	Discharge after recovery
Probable Vertical Transmission of SARS-CoV-2 Infection	Demirjian et al (2020) (29)	1	Term infant	5 at 1 minute<br>9 at 5 minutes<br>9 at 10 minutes	Fever<br>Tachypnea<br>Coryza	Discharge after recovery
First case of neonatal infection due to SARS-CoV-2 in Spain	Díaz et al (2020) (30)	1	Term infant	7 at 1 minute<br>9 at 5 minutes	Respiratory distress<br>Hyperpnea	Discharge after recovery
Possible Vertical Transmission of SARS-CoV-2 From an Infected Mother to Her Newborn	Dong et al (2020) (31)	1	Premature	9 at 1 minute<br>10 at 5 minutes	Asymptomatic	Discharge after recovery

Neonatal Coronavirus 2019 (COVID-19) Infection: A Case Report and Review of Literature	Dumpa et al (2020) (32)	1	Term infant	9 at 1 minute<br>9 at 5 minutes	Fever<br>Intolerance to feeding	Discharge after recovery
COVID-19 Virus in a 6-Day-Old Girl Neonate: A Case Report	Eghbalian et al (2020) (33)	1	Term infant	9 at 1 minute<br>10 at 5 minutes	Asymptomatic	Discharge after recovery
A Neonate With Vertical Transmission of COVID-19 and Acute Respiratory Failure	Farmer (2021) (34)	1	Premature	Not reported	Respiratory Distress<br>Acute respiratory failure<br>Pulmonary hypertension	Discharge after recovery
IN-UTERO MOTHER-TO-CHILD SARS-CoV-2 TRANSMISSION: viral detection and fetal immune response	Fenizia et al (2020) (35)	2	**Neonate 1:**<br>Premature<br>**Neonate 2:**<br>Term infant	**Neonate 1:**<br>9 at 1 minute<br>10 at 5 minutes<br>**Neonate 2:**<br>9 at 1 minute<br>10 at 5 minutes	**Neonate 1:**<br>Asymptomatic<br>**Neonate 2:**<br>asymptomatic	Discharge after recovery
Neonatal apnea as initial manifestation of SARS-CoV-2 infection	Gonzales Brabin et al (2020) (36)	1	Term infant	Not reported	Apnea<br>Hypotonia<br>Respiratory distress	Discharge after recovery
Rapid systematic review of neonatal COVID-19 including a case of	Gordon et al (2020) (37)	1	Premature	Not reported	Asymptomatic	Discharge after recovery

presumed vertical transmission						
Point-of-care lung ultrasound in three neonates with COVID-19	Gregorio-Hernández et al (2020) (38)	3	**Neonate 1:** Premature **Neonate 2:** Term infant **Neonate 3:** Term infant	Not reported	**Neonate 1:** Respiratory distress **Neonate 2:** Respiratory distress **Neonate 3:** Respiratory distress	Discharge after recovery
Sequential Analysis of Viral Load in a Neonate and Her Mother Infected With Severe Acute Respiratory Syndrome Coronavirus 2	Han et al (2020) (39)	1	Term infant	Not reported	Cough Fever Vomiting Tachycardia Coryza	Discharged after recovery
Severe Acute Respiratory Syndrome Coronavirus 2 (SARS-CoV-2) Vertical Transmission in Neonates Born to Mothers With Coronavirus Disease 2019 (COVID-19) Pneumonia	Hu et al (2020) (40)	1	Term infant	8 at 1 minute 9 at 5 minutes	Asymptomatic	Not reported
A neonate born to an infected COVID-19 mother was tested positive just 24 hours after its birth	Huseynova *et al* (2020) (41)	1	Term infant	8 at 1 minute 9 at 5 minutes	Asymptomatic	Discharge after recovery

Probable congenital SARS-CoV-2 infection in a neonate born to a woman with active SARS-CoV-2 infection	Kirtsman et al (2020) (42)	1	Premature	9 at 1 minute<br>9 at 5 minutes	Hypothermia<br>Intolerance to feeding<br>Hypoglycemic episodes	Discharge after recovery
Early-onset symptomatic neonatal COVID-19 infection with high probability of vertical transmission	Kulkarni et al (2021) (43)	1	Term infant	6 at 1 minute<br>9 at 5 minutes	Fever<br>Intolerance to feeding	Discharge after recovery
Respiratory Failure in an Extremely Premature Neonate with COVID-19	Kumar *et al* (2021) (44)	1	Premature	1 at 1 minute<br>5 at 5 minutes	Apnea<br>Bradycardia<br>Respiratory distress	Discharge after recovery
Neonatal Early-Onset Infection With SARS-CoV-2 in a Newborn Presenting With Encephalitic Symptoms	Lorenz et al (2020) (45)	1	Term infant	9 at 1 minute<br>9 at 5 minutes	Lethargy<br>Fever<br>Irritability<br>Hyper-excitability<br>Respiratory distress<br>Cough<br>Hypopnea	Discharge after recovery
MR Imaging Findings in a Neonate With COVID - 19-Associated Encephalitis	Martin *et al* (2021) (46)	1	Term infant	Not reported	Seizures<br>Encephalitis<br>Hypoxia	Discharge after recovery
Association Between Mode of Delivery Among Pregnant Women With COVID-19 and Maternal and Neonatal Outcomes in Spain	Martínez-Perez et al (2020) (47)	3	**Neonate 1:**<br>Term infant<br>**Neonate 2:**<br>Term infant	Not reported	**Neonate 1:**<br>Asymptomatic<br>**Neonate 2:**<br>Asymptomatic	Discharge after recovery

			**Neonate 3:** Term infant		**Neonate 3:** Asymptomatic	
Possible Coronavirus Disease 2019 Pandemic and Pregnancy: Vertical Transmission Is Not Excluded	Marzollo et al (2020) (48)	1	Term infant	9 at 1 minute<br>10 at 5 minutes	Intolerance to feeding<br>Abdominal distension<br>Hyper-excitability	Discharge after recovery
Coronavirus Disease 2019 in Newborns and Very Young Infants: a Series of Six Patients in France	Meslin et al (2019) (49)	4	**Neonate 1:** Term infant<br>**Neonate 2:** Term infant<br>**Neonate 3:** Term infant<br>**Neonate 4:** Term infant	Not reported	**Neonate 1:** Fever<br>Coryza<br>**Neonate 2:** Fever<br>**Neonate 3:** Fever<br>**Neonate 4:** Fever	Discharge after recovery

COVID-19 Infection in Iranian Newborns and their Mothers: a Case Series	Mohagheghi et al (2021) (50)	13	**Neonate 1:**<br>Term infant<br>**Neonate 2:**<br>Premature<br>**Neonate 3:**<br>Premature<br>**Neonate 4:**<br>Term infant<br>**Neonate 5:**<br>Term infant<br>**Neonate 6:**<br>Premature<br>**Neonate 7:**<br>Premature<br>**Neonate 8:**<br>Premature<br>**Neonate 9:**<br>Term infant<br>**Neonate 10:**<br>Premature<br>**Neonate 11:**<br>Premature<br>**Neonate 12:**<br>Premature<br>**Neonate 13:**<br>Term infant	**Neonate 1:**<br>4 at 1 minute<br>8 at 5 minutes<br>**Neonate 2:**<br>4 at 1 minute<br>8 at 5 minutes<br>**Neonate 3:**<br>7 at 1 minute<br>8 at 5 minutes<br>**Neonate 4:**<br>9 at 1 minute<br>10 at 5 minutes<br>**Neonate 5:**<br>8 at 1 minute<br>9 at 5 minutes<br>**Neonate 6:**<br>8 at 1 minute<br>9 at 5 minutes<br>**Neonate 7:**<br>Not reported	**Neonate 1:**<br>Cough<br>Lethargy<br>Respiratory distress<br>**Neonate 2:**<br>Lethargy<br>Respiratory distress<br>**Neonate 3:**<br>Respiratory distress<br>**Neonate 4:**<br>Respiratory distress<br>**Neonate 5:**<br>Respiratory. distress<br>**Neonate 6:**<br>Respiratory distress<br>**Neonate 7:**<br>Respiratory distress<br>**Neonate 8:**<br>Respiratory distress<br>**Neonate 9:**<br>Fever<br>Intolerance to feeding<br>Lethargy	Discharge after recovery (=11)<br>Death (neonate 7 and 12)

				**Neonate 8:**<br>3 at 1 minute<br>7 at 5 minutes<br>**Neonate 9:**<br>9 at 1 minute<br>10 at 5 minutes<br>**Neonate 10:**<br>7 at 1 minute<br>9 at 5 minutes<br>**Neonate 11:**<br>9 at 1 minute<br>10 at 5 minutes<br>**Neonate 12:**<br>2 at 1 minute<br>5 at 5 minutes<br>**Neonate 13:**<br>9 at 1 minute<br>13 at 5 minutes	**Neonate 10:**<br>Respiratory distress<br>**Neonate 11:**<br>Respiratory distress<br>**Neonate 12:**<br>Lethargy<br>Respiratory distress<br>**Neonate 13:**<br>Respiratory distress	

COVID-19 in Neonates and Infants: Progression and Recovery	Ng et al (2020)(51)	1	Premature	Not reported	Intolerance to feeding<br>Lethargy<br>Jaundice<br>Apnea	Discharge after recovery
A multicenter study on epidemiological and clinical characteristics of 125 newborns born to women infected with COVID-19 by Turkish Neonatal Society	Oncel et al (2021) (52)	4	**Neonate 1:**<br>Premature<br>**Neonate 2:**<br>Premature<br>**Neonate 3:**<br>Term infant<br>**Neonate 4:**<br>Term infant	**Neonate 1:**<br>2 at 1 minute<br>5 at 5 minutes<br>**Neonate 2:**<br>7 at 1 minute<br>8 at 5 minutes<br>**Neonate 3:**<br>8 at 1 minute<br>9 at 5 minutes<br>**Neonate 4:**<br>8 at 1 minute<br>9 at 5 minutes	**Neonate 1:**<br>Tachypnea<br>Intolerance to feeding<br>**Neonate 2:**<br>Tachypnea<br>Fever<br>Cough<br>**Neonate 3:**<br>Asymptomatic<br>**Neonate 4:**<br>Tachypnea	Discharge after recovery

Neonates with Covid-19 infection: Is there any different treatment process?	Pakdel *et al* (2022) (53)	17	**Neonate 1:**<br>Term infant<br>**Neonate 2:**<br>Term infant<br>**Neonate 3:**<br>Term infant<br>**Neonate 4:**<br>Term infant<br>**Neonate 5:**<br>Premature<br>**Neonate 6:**<br>Term infant<br>**Neonate 7:**<br>Premature<br>**Neonate 8:**<br>Premature<br>**Neonate 9:**<br>Premature<br>**Neonate 10:**<br>Term infant<br>**Neonate 11:**<br>Premature<br>**Neonate 12:**<br>Premature<br>**Neonate 13:**<br>Term infant<br>**Neonate 14:**<br>Term infant<br>**Neonate 15:**	**Neonate 1:**<br>8 at 1 minute<br>9 at 5 minutes<br>**Neonate 2:**<br>9 at 1 minute<br>10 at 5 minutes<br>**Neonate 3:**<br>9 at 1 minute<br>10 at 5 minutes<br>**Neonate 4:**<br>9 at 1 minute<br>9 at 5 minutes<br>**Neonate 5:**<br>8 at 1 minute<br>8 at 5 minutes<br>**Neonate 6:**<br>9 at 1 minute<br>10 at 5 minutes<br>**Neonate 7:**<br>8 at 1 minute	**Neonate 1:**<br>Tachypnea<br>Respiratory distress<br>Seizure<br>**Neonate 2:**<br>Fever<br>Intolerance to feeding<br>**Neonate 3:**<br>Tachypnea<br>Respiratory distress<br>Hypotonia<br>**Neonate 4:**<br>Fever<br>Vomiting<br>Respiratory distress<br>**Neonate 5:**<br>Tachypnea<br>Respiratory distress<br>Seizure<br>**Neonate 6:**<br>Fever<br>Intolerance to feeding<br>Hypotonia<br>**Neonate 7:**<br>Asymptomatic	Discharge after recovery (=16)<br>Death (=1)<br>(Neonate 11)

			Term infant<br>**Neonate 16:**<br>Term infant<br>**Neonate 17:**<br>Term infant	10 at 5 minutes<br>**Neonate 8:**<br>8 at 1 minute<br>10 at 5 minutes<br>**Neonate 9:**<br>4 at 1 minute<br>8 at 5 minutes<br>**Neonate 10:**<br>7 at 1 minute<br>10 at 5 minutes<br>**Neonate 11:**<br>6 at 1 minute<br>8 at 5 minutes<br>**Neonate 12:**<br>5 at 1 minute<br>8 at 5 minutes<br>**Neonate 13:**<br>9 at 1 minute<br>10 at 5 minutes<br>**Neonate 14:**	**Neonate 8:**<br>Fever<br>**Neonate 9:**<br>Tachypnea<br>Respiratory distress<br>Apnea<br>**Neonate 10:**<br>Tachypnea<br>Respiratory distress<br>Abdominal distension<br>Vomiting<br>**Neonate 11:**<br>Tachypnea<br>Respiratory distress<br>Abdominal distension<br>Apnea<br>Seizure<br>Necrotizing enterocolitis<br>**Neonate 12:**<br>Tachypnea<br>Respiratory distress<br>Abdominal distension<br>Vomiting	

				8 at 1 minute<br>10 at 5 minutes<br>**Neonate 15:**<br>9 at 1 minute<br>10 at 5 minutes<br>**Neonate 16:**<br>9 at 1 minute<br>10 at 5 minutes<br>**Neonate 17:**<br>9 at 1 minute<br>10 at 5 minutes	Apnea<br>Seizure<br>Necrotizing enterocolitis<br>**Neonate 13:**<br>Hypotonia<br>**Neonate 14:**<br>Cough<br>Intolerance to feeding<br>**Neonate 15:**<br>Fever<br>Intolerance to feeding<br>**Neonate 16:**<br>Tachypnea<br>Respiratory distress<br>**Neonate 17:**<br>Fever<br>Tachypnea<br>Respiratory distress	

Vertical transmission of coronavirus disease 2019: severe acute respiratory syndrome coronavirus 2 RNA on the fetal side of the placenta in pregnancies with coronavirus disease 2019–positive mothers and neonates at birth	Patanè et al (2020) (54)	2	**Neonate 1:**<br>Premature<br>**Neonate 2:**<br>Premature	**Neonate 1:**<br>9 at 1 minute<br>10 at 5 minutes<br>**Neonate 2:**<br>9 at 1 minute<br>10 at 5 minutes	**Neonate 1**:<br>Intolerance to feeding<br>**Neonate 2:**<br>Intolerance to feeding	Discharge after delivery
Newborns of COVID-19 mothers: short-term outcomes of colocating and breastfeeding from the pandemic's epicenter	Patil et al (2020) (55)	3	**Neonate 1:**<br>Premature<br>**Neonate 2:**<br>Premature<br>**Neonate 3:**<br>Premature	Not reported	**Neonate 1:**<br>Asymptomatic<br>**Neonate 2:**<br>Asymptomatic<br>**Neonate 3:**<br>Asymptomatic	Discharge after recovery
COVID-19 in a 26-week preterm neonate.	Piersigilli et al (2020) (56)	1	Premature	5 at 1 minute<br>8 at 5 minutes	Respiratory distress	Discharge after recovery
A Case Report of Neonatal Acute Respiratory Failure Due to Severe Acute Respiratory Syndrome Coronavirus-2	Precit et al (2020) (57)	1	Term infant	Not reported	Coryza<br>Respiratory distress<br>Lethargy	Discharge after recovery
Vertical Transmission of COVID-19: A Case Report and Review of Literature	Thapa et al (2021) (58)	1	Term infant	"Normal"	Tachypnea	Discharge after recovery

Mother and neonate suffering from COVID-19 infection. Is there any risk of vertical transmission?	Urban et al (2021) (59)	1	Term infant	10 at 1 minute	Fever<br>Irritability	Discharge after recovery
SARS-CoV-2 infection with gastrointestinal symptoms as the first manifestation in a neonate	Wang et al (2020) (60)	1	Term infant	Not reported	Cough<br>Fever<br>Vomiting<br>Intolerance to feeding<br>Irritability	Discharge after recovery
A case report of neonatal COVID-19 infection in China	Wang et al (2020) (61)	1	Term infant	8 at 1 minute<br>9 at 5 minutes	Asymptomatic	Discharge after recovery
Clinical features and obstetric and neonatal outcomes of pregnant patients with COVID-19 in Wuhan, China: a retrospective, single-centre, descriptive study	Yu et al (2020) (62)	1	Term infant	"Normal"	Respiratory distress	Discharge after recovery
